# Assessing the impact of the COVID-19 pandemic on childhood vaccine uptake with administrative data

**DOI:** 10.1016/j.ssmph.2024.101657

**Published:** 2024-03-28

**Authors:** Leon Iusitini, Gail Pacheco, Thomas Schober

**Affiliations:** New Zealand Policy Research Institute, Auckland University of Technology, New Zealand

**Keywords:** Childhood vaccine uptake, Immunisation, COVID-19 pandemic

## Abstract

This study examines the impact of the COVID-19 pandemic on childhood vaccination coverage in New Zealand using population-wide administrative data. For each immunisation event from ages 6 weeks to 4 years, we compare vaccine uptake of children who became eligible for immunisation during the pandemic to earlier-born cohorts whose immunisations were due before the pandemic. We find that the initial phase of the pandemic had, on average, small or nil effects on timely immunisation at the four infancy events, but a large effect at the 4-year event of −15 percentage points. Nine months after eligibility, catch-up among the pandemic-affected cohorts was largely achieved for the infancy immunisations, but 4-year coverage remained 6 percentage points below pre-pandemic levels. Vaccine uptake at 4 years initially dropped most among children of European ethnicity and of non-beneficiary parents but catch-up quickly surpassed their Māori, Pacific, and beneficiary counterparts for whom sizeable gaps in coverage below pre-pandemic levels remained at the end of our observation period. The pandemic thus widened pre-existing inequalities in immunisation coverage.

## Highlights

• Decrease in timely immunisation during the initial phase of the COVID-19 pandemic more pronounced in older children.
• Only partial catch-up during the course of the pandemic.
• Large differences related to ethnicity and socioeconomic status.
• Widening of pre-existing inequalities in immunisation coverage.

## 1 Introduction

The COVID-19 pandemic has posed a threat to the uptake of routine childhood immunisations, increasing the risk of outbreaks of vaccine-preventable diseases. Across the globe, the delivery of immunisation programmes was disrupted due to a range of factors including redeployment of healthcare workers to pandemic response activities, reduced vaccine supplies, fear of COVID-19 exposure, travel restrictions, and misinformation (Shet et al., 2022). As a consequence of this disruption, current international evidence has predominantly found that the pandemic's initial impact on childhood immunisation uptake was negative with immediate declines in coverage observed around the world (Lassi et al., 2021; Ota et al., 2021). The success of immunisation programmes relies on high coverage rates both at national and sub-national levels to provide direct protection for individuals and indirect protection for communities through herd immunity.

Our research aims to estimate the impact of the pandemic on paediatric vaccination coverage in New Zealand using national immunisation registry data linked to population-wide administrative information. Our analysis compares the vaccination coverage of New Zealand children who became eligible for immunisation during the pandemic with coverage among earlier-born cohorts whose immunisations were due before the pandemic. Our use of linked integrated data at a nationwide level permits estimation of the initial impact and consequent catch-up rate for different vaccination events (between birth and age four) for affected cohorts, as well as a wide range of heterogeneity analyses to ascertain differential impacts on specific population groups. Furthermore, most of the existing literature has focused on particular regions, provinces, or jurisdictions, whereas the linked administrative data used in this study allows analysis across the entire population of children born in New Zealand.

New Zealand's National Immunisation Schedule^1^ is a series of publicly-funded vaccines available to New Zealanders from six weeks to 65 years of age and in pregnancy (free for children aged under 18). The National Immunisation Register (NIR) records the immunisation details of New Zealand children and selected adult vaccines since 2005 (Ministry of Health, 2023). The NIR information system thus permits monitoring of the uptake of the National Immunisation Schedule.

New Zealand recorded its first case of COVID-19 on 28 February 2020. Earlier that month, the government had introduced border controls (entry restrictions and quarantine of arriving travellers) and from 21 March 2020 a four-level alert system was adopted with the aim of eliminating COVID-19 which had begun to spread in the community. The highest alert level (Level 4) involved a nationwide lockdown or stay-at-home order in place from 26 March to 27 April 2020. Partial lockdown at Alert Level 3 continued until 13 May 2020. The elimination strategy was initially successful, with no community transmission detected from early May and zero new cases sustained for over 100 days thereafter (Baker et al., 2020). This ended with an outbreak in Auckland (New Zealand's most-populous and main gateway city) in August 2020, at which point a regional lockdown at Level 3 was implemented in Auckland. Throughout the remainder of 2020 and most of 2021 there were a series of lockdowns which varied in location and stringency depending on the nature of the outbreak and extent of community transmission at the time. While lockdowns substantially reduced health service utilisation across both hospital and general practice activity (Ministry of Health, 2020), the delivery of immunisation services in primary care (such as routine paediatric vaccinations) was intended to continue as an ‘essential service’ throughout all alert levels (Ministry of Health, 2021).

This study adds to the growing literature on the impacts of the pandemic on childhood vaccination coverage. International evidence initially relied on proxy information based on the number of vaccines doses ordered or administered by physicians in early 2020 to estimate the pandemic's impact - see Aizawa et al. (2021) for Japan, Santoli et al. (2020) for the U.S., and McDonald et al. (2020) for England. The handful of retrospective cohort studies analysing vaccine *coverage* (rather than counts) based on immunisation registry data have mostly found negative impacts at the start of the pandemic (for example, Ackerson et al. (2021), Bode et al. (2021), DeSilva et al. (2022), and Ji et al. (2022)). Interestingly, a few studies have found *positive* impacts on paediatric immunisation uptake in some countries - see Martínez-Marcos et al. (2023) for Spain and McQuaid et al. (2022) for Scotland.

Evidence is also limited on the extent of catch-up in vaccination coverage beyond the initial lockdowns and on the differential impact across population groups to better understand equity implications. On the latter, the evidence that does exist is mixed - Ackerson et al. find that the decline in coverage was larger, and recovery during the ‘reopening period’ of the pandemic was weaker, among Black children in the US compared to non-Black children, whereas DeSilva et al. (2022) find that pre-pandemic racial differences in coverage in the US were mostly preserved (not exacerbated) during 2020.^2^

Our study's contribution to the literature is three-fold: (i) A detailed assessment of the initial impact of the pandemic, and the extent of vaccination catch-up, in a strict policy response environment (New Zealand's stringent lockdown policies across 2020 and 2021 present a useful case study for investigating the pandemic's impact on routine paediatric vaccination uptake and catch-up); (ii) A population-wide analysis, given much of the prior international literature is focused on a particular region or on selective or unrepresentative population groups; and (iii) Investigation of heterogeneous impacts by different socio-demographic characteristics including with controls for potentially confounding characteristics due to the ability to link the NIR to other administrative data sources. The remainder of this paper consists of: Section 2 describes the integrated data sources; Section 3 presents descriptive trends in vaccination coverage for different immunisation events between birth and age four; Section 4 details the empirical strategy to analyse the effect of the pandemic on vaccine uptake (both average effects and heterogeneous effects); and Section 5 concludes.

## 2 Data

We use data from the Integrated Data Infrastructure (IDI), a large research database managed by Stats NZ. It holds micro-data from various government agencies, organisations, and surveys with longitudinal information on education, income, health and other life events that can be linked at the individual level (Stats NZ, 2020b). The IDI includes administrative data from the NIR which we use to measure vaccine uptake. It provides data on immunisation events such as the date and type of administered vaccines and is considered to be of high data accuracy compared with international equivalents, especially for vaccines covered by the National Immunisation Schedule (Chisholm et al., 2021).

Our population of interest is constructed using data on births registered in New Zealand from the Department of Internal Affairs (DIA). We restrict the analysis to children living in New Zealand and exclude those who have died or moved overseas, using information on deaths and international travel. We also use the birth register to link children to their parents to examine families’ characteristics.^3^

Information on child and parental characteristics come from various data sources. Child sex, ethnicity, and place of residence is derived from the IDI central tables collated by Stats NZ. Regarding ethnicity, we allocated each child to a single ethnic group using the Ministry of Health (2017) prioritisation for level 1 codes (priority order: Māori, Pacific Peoples, Asian, Middle Eastern/Latin American/African, Other Ethnicity, European). Inland Revenue provides income tax data. For each child, we identify the main earner of the family by using the maximum of mother's and father's earnings (income from salaries and wages) in the calendar year before birth. We then characterise parental earnings as low (below the 33rd percentile), medium, or high (above the 66th percentile).

Information on parents' education comes from the 2018 census, which we use to indicate if any of a child's parents has a bachelor's degree or higher. Information on whether a child has an overseas-born parent comes from the administrative population census, which itself combines data from the census, birth registrations, visa applications and border movements (Stats NZ, 2021). We use the New Zealand Deprivation Index 2018 (NZDep2018) to measure the level of socioeconomic deprivation of the neighbourhood and distinguish areas with low (levels 1–3), medium (4–7), and high (8–10) deprivation.

## 3 Trends in childhood vaccine uptake

**Fig. 1:**
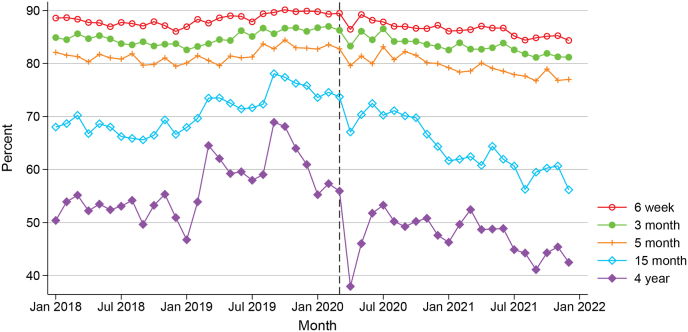
Share of children fully immunised within one month of becoming eligible, for different immunisation events over time (vertical line indicates the onset of the pandemic in March 2020).

Fig. 1 summarises the share of fully immunised children within one month of becoming eligible for all analysed immunisation events (6 weeks, 3, 5, and 15 months, and 4 years). We select children who reach a specific age in each calendar month and assess if they receive all recommended vaccines up to that age to determine their vaccination status.^4^ Therefore, Fig. 1 is based on a different population cohort at each time point.

There are a number of notable trends from this descriptive figure. First, it shows that, irrespective of year of observation, the share of children immunised on time is lower for later immunisation events. At the beginning of the analysis period in 2018, around 80–90 % of children receive the scheduled vaccines in the first year of life within one month of becoming eligible. This share drops to below 70 % for the 15 month event, and to below 60 % for the 4 year event. Similar patterns are observed in later years.

Second, it is worth assessing trends in vaccine coverage before the pandemic. For the first three immunisation events (6 weeks, 3 and 5 month vaccines), there is not much variation pre-pandemic; whereas for the later ages, there is greater volatility in uptake, with some evidence of a declining trend in the last few months of 2019. Nevertheless, the average vaccine uptake for both the 15 month and 4 year events at the start of 2020 was still slightly higher than the corresponding uptake at the start of 2018.

Finally, once the pandemic hit, we see a visible drop in vaccine uptake for all events. The drop is more pronounced among older children. For the 4 year event, the share of fully immunised children declines from 56 % in March 2020 to 38 % in April 2020. Uptake bounces back in the subsequent few months, but over the entire course of 2020 and 2021, Fig. 1 indicates a steady downward trend in on-time vaccinations for all immunisation events (particularly among older children again).

## 4 Effects of the pandemic

### 4.1 Empirical strategy

To analyse the effect of the pandemic on vaccine uptake, we focus on children who become eligible for immunisation during the pandemic. Section 3 above reveals large drops in immunisations in the first months of the pandemic, when the country went into a national lockdown. For each immunisation event, we therefore analyse the vaccination status of children who reach the age at which immunisation is recommended in March, April, or May 2020. For example, for the 4 year immunisation event, the cohort of affected children consists of children born in March to May 2016. We contrast vaccine uptake of affected children with a cohort of unaffected children born one year earlier. For the 4 year event, this group consists of children born in March to May 2015. Table 1 provides and overview of the selected cohorts by immunisation event. Note also that the policy contexts at birth, in terms of vaccines recommended on the immunisation schedule, are the same for both the affected and unaffected cohorts.

**Table 1:** Birth months of selected children.

Immunisation event	Affected cohort	Unaffected cohort
4 year	March–May 2016	March–May 2015
15 month	Dec 2018–Feb 2019	Dec 2017–Feb 2018
5 month	Oct–Dec 2019	Oct–Dec 2018
3 month	Dec 2019–Feb 2020	Dec 2018–Feb 2019
6 week	Jan–March 2020	Jan–March 2019

Notes: This table shows the birth months of children in the affected and unaffected cohorts used to estimate the effects of the COVID-19 pandemic on vaccine uptake.

We compare vaccination status between these groups at each month as children grow older, to precisely analyse gaps in uptake and potential catch-up over time. To do so, we estimate the following linear probability model:

(1) $$v_{i}=\alpha +\beta A_{i}+\gamma X_{i}+\epsilon_{i},$$

where *v*_*i*_ is a dummy variable indicating if child *i* has received the recommended vaccines, *A*_*i*_ is a dummy variable indicating if the child belongs to the group of affected children, and *X*_*i*_ is a vector of control variables. For the recommended vaccines, we follow the relevant immunisation schedule (provided in Table A1 in Appendix A) and consider children to be fully vaccinated if they have received the recommended vaccines for the respective immunisation event. We estimate equation (1) at nine different points in time for each immunisation event, depending on the age of the children. For the 4 year immunisation event, for example, we follow children at each month from age 49 months–57 months.^5^ In baseline regressions, control variables include child's sex and dummy variables for the calendar month of birth. In sensitivity checks, we additionally control for region of residence, child's ethnicity, parental earnings level, parental marital status, and overseas-born parents. We then provide two types of heterogeneity analysis. First, we estimate equation (1) separately for different sub-samples of the population. Second, we present average marginal effects derived from regressions where we jointly include all analysed child and parental characteristics.

Coefficient *β* provides an estimate of the effect of the pandemic on vaccine uptake. The underlying assumption is that the group of affected children would have behaved similarly to the earlier-born cohort in the absence of the COVID-19 pandemic. Any differences in uptake can then be attributed to the pandemic. Similar strategies have been used, for example, to analyse effects of measles outbreaks (Schober, 2020) and influenza pandemics (Almond & Mazumder, 2005; Fletcher, 2018).

While we cannot directly test the assumption that children affected by the pandemic would have behaved similar to those who were born earlier, we can compare their characteristics to assess any observable differences between cohorts. Table 2 shows that child and parental characteristics are very similar in both groups at the 4 year immunisation event. Observable differences are either statistically insignificant or small. The affected cohort has a slightly larger share of Asian children and has parents with somewhat higher earnings and lower benefit dependency.^6^ We control for background characteristics in sensitivity checks below to test if these differences affect our estimates.

**Table 2:** Characteristics of affected and unaffected children at 4 year immunisation event (in %).

	(1)	(2)	(3)	(4)
	Covid-Affected	Covid-Unaffected	Difference	p-value
Female	48.3	48.4	0.1	0.853
European	43.6	45.3	1.7	0.005
Māori	29.8	30.0	0.2	0.758
Pacific People	9.3	9.5	0.2	0.628
Asian	14.7	12.8	−1.9	0.000
Information on mother	99.0	99.1	0.1	0.488
Information on father	94.2	93.9	−0.3	0.280
Characteristics of parents				
Born overseas	36.1	34.8	−1.3	0.032
Married	51.5	50.7	−0.8	0.174
Benefit receipt	25.5	26.8	1.3	0.014
Bachelor's degree or higher	40.3	38.8	−1.5	0.013
Low earnings	35.6	37.8	2.1	0.000
Medium earnings	32.9	33.2	0.3	0.647
High earnings	31.4	29.0	−2.4	0.000
Neighbourhood deprivation level				
Low deprivation	23.3	23.4	0.1	0.926
Medium deprivation	35.8	36.1	0.4	0.517
High deprivation	40.9	40.5	−0.4	0.476

Notes: This table compares average characteristics of children affected (Column 1) and unaffected (2) by the COVID-19 pandemic. Column 3 shows the difference between groups, Column 4 shows the p-value testing the equality of the two means. The number of observations is 12,618 for parents' education level and 13,164 for all other characteristics. Observation counts are randomly rounded to base 3 in accordance with Stats NZ confidentiality rules.

**Table 3:** Effects of the COVID-19 pandemic on childhood vaccine uptake.

	1 month after eligibility			9 months after eligibility			N
	Mean	Estimate	S.E.	Mean	Estimate	S.E.	
	(1)	(2)	(3)	(4)	(5)	(6)	(7)
*Panel A: Baseline specification*							
6 week event	0.92	−0.00	(0.003)	0.93	−0.00	(0.003)	27504
3 month event	0.85	−0.01	(0.004)	0.89	−0.01	(0.004)	27039
5 month event	0.84	−0.01**	(0.005)	0.94	−0.01***	(0.003)	27567
15 month event	0.75	−0.03***	(0.005)	0.89	−0.01	(0.004)	26478
4 year event	0.61	−0.15***	(0.006)	0.91	−0.06***	(0.004)	26139
*Panel B: Additional control variables*							
6 week event	0.92	−0.01	(0.004)	0.93	−0.00	(0.004)	17409
3 month event	0.85	−0.01*	(0.004)	0.89	−0.01*	(0.004)	25998
5 month event	0.84	−0.02***	(0.004)	0.94	−0.02***	(0.003)	26496
15 month event	0.75	−0.03***	(0.005)	0.89	−0.01*	(0.004)	25515
4 year event	0.61	−0.15***	(0.006)	0.91	−0.07***	(0.004)	25473

Notes: This table summarises the average effects of the COVID-19 pandemic on vaccine uptake. Each estimate represents the results from a separate regression for different immunisation events 1 and 9 months after children become eligible. Columns 1 and 4 show the mean of the unaffected cohort, columns 2 and 5 the point estimate, columns 3 and 6 robust standard error, and column 7 the number of children in the regression. Panel A includes controls for child's calendar month of birth and sex, Panel B adds control variables for region of residence, child's ethnicity, parental earnings level, parental marital status, and overseas-born parents. *p < 0.05, **p < 0.01, ***p < 0.001.

**Table 4:** Child characteristics and vaccine uptake at the 4 year immunisation event.

	49 months			57 months			N
	Mean	Estimate	S.E.	Mean	Estimate	S.E.	
	(1)	(2)	(3)	(4)	(5)	(6)	(7)
*Panel A: Ethnicity*							
European	0.67	−0.18***	(0.01)	0.93	−0.04***	(0.01)	11610
Māori	0.45	−0.13***	(0.01)	0.84	−0.12***	(0.01)	7818
Pacific People	0.55	−0.12***	(0.02)	0.91	−0.11***	(0.01)	2454
Asian	0.78	−0.12***	(0.01)	0.97	−0.02**	(0.01)	3594
*Panel B: Sex*							
Male	0.61	−0.15***	(0.01)	0.91	−0.07***	(0.01)	13494
Female	0.61	−0.15***	(0.01)	0.90	−0.06***	(0.01)	12648
*Panel C: Birth order*							
First born	0.63	−0.15***	(0.01)	0.91	−0.05***	(0.01)	13563
Second born	0.63	−0.16***	(0.01)	0.92	−0.05***	(0.01)	7965
Third born	0.54	−0.14***	(0.02)	0.90	−0.09***	(0.01)	3012
Fourth born	0.47	−0.17***	(0.03)	0.88	−0.14***	(0.03)	945

Notes: This table summarises the heterogeneous effects of the COVID-19 pandemic on the 4 year immunisation event at age 49 months (mean of the unaffected cohort in column 1, point estimate in column 2, robust standard error in column 3) and 57 months (columns 4 to 6). Each estimate represents the results from a separate regression for a subgroup of the sample indicated on the left. All regressions include controls for the child's calendar month of birth and sex. *p < 0.05, **p < 0.01, ***p < 0.001.

**Table 5:** Parental characteristics and vaccine uptake at the 4 year immunisation event.

	49 months			57 months			N
	Mean	Estimate	S.E.	Mean	Estimate	S.E.	
	(1)	(2)	(3)	(4)	(5)	(6)	(7)
*Panel A: Earnings*							
Low earnings	0.51	−0.14***	(0.01)	0.85	−0.10***	(0.01)	9594
Medium earnings	0.63	−0.14***	(0.01)	0.93	−0.06***	(0.01)	8637
High earnings	0.71	−0.18***	(0.01)	0.96	−0.04***	(0.01)	7908
*Panel B: Benefit receipt*							
No benefit receipt	0.67	−0.16***	(0.01)	0.94	−0.04***	(0.00)	19311
Benefit receipt	0.44	−0.13***	(0.01)	0.83	−0.13***	(0.01)	6831
*Panel C: Education level*							
No degree	0.56	−0.14***	(0.01)	0.89	−0.09***	(0.01)	15147
Bachelor's degree or higher	0.70	−0.16***	(0.01)	0.95	−0.03***	(0.01)	9906
*Panel D: Marital status*							
Unmarried parents	0.53	−0.14***	(0.01)	0.87	−0.10***	(0.01)	12786
Married parents	0.69	−0.15***	(0.01)	0.94	−0.04***	(0.00)	13353
*Panel E: Birth place*							
Born in NZ	0.57	−0.16***	(0.01)	0.89	−0.08***	(0.01)	16869
Born overseas	0.68	−0.13***	(0.01)	0.94	−0.04***	(0.01)	9270
*Panel F: Neighbourhood deprivation level*							
Low deprivation	0.69	−0.18***	(0.01)	0.95	−0.04***	(0.01)	6108
Medium deprivation	0.63	−0.14***	(0.01)	0.91	−0.04***	(0.01)	9396
High deprivation	0.54	−0.14***	(0.01)	0.88	−0.10***	(0.01)	10635

Notes: This table summarises the heterogeneous effects of the COVID-19 pandemic on the 4 year immunisation event at age 49 months (mean of the unaffected cohort in column 1, point estimate in column 2, robust standard error in column 3) and 57 months (columns 4 to 6). Each estimate represents the results from a separate regression for a subgroup of the sample indicated on the left. All regressions include controls for the child's calendar month of birth and sex. *p < 0.05, **p < 0.01, ***p < 0.001.

Tables A2, A3, A4, and A5 in Appendix A compare characteristics for the remaining immunisation events. Overall, we find similar distributions of characteristics between groups which support the identifying assumption. The similarity is also plausible because we always compare children who are only born one year apart, and, at the population level, it can be expected that the characteristics of children born in New Zealand change only slowly over time.

### 4.2 Average effects

**Fig. 2:**
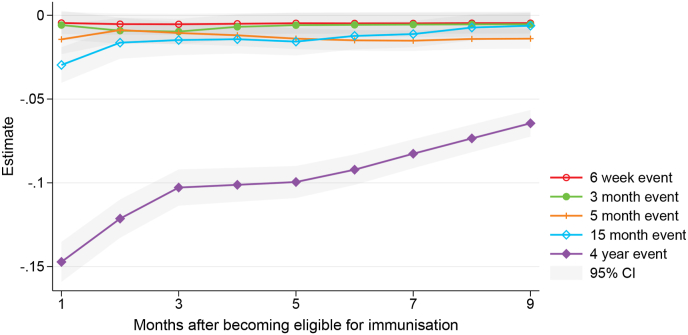
Effect of the COVID-19 pandemic on childhood vaccine uptake at different immunisation events.

Fig. 2 summarises the results of estimating equation (1) for different immunisation events, Table 3 Panel A shows the corresponding regression output at two time periods—1 month and 9 months after children become eligible for immunisation. The pandemic had large effects on timely vaccination at the 4 year event. It led to a 15 percentage point (pp) reduction in the share of fully immunised children 1 month after they became eligible (at 49 months of age). The affected group of children managed to partially catch-up during the course of the pandemic, however, 9 months after eligibility (at 57 months of age) uptake was still 6 pp lower.

Effects on earlier immunisation events (the four infancy events from 6 weeks to 15 months) are smaller. At the 15 month event, the pandemic led to a 3 pp reduction in uptake 1 month after eligibility, and there is no statistically significant effect 9 months after eligibility. At the 5 month event, there is a 1 pp difference at the start and at the end of the observed period, while there are no statistically significant effects at the 3 month and 6 week events. The larger effects at later immunisation events in the regression results are consistent with the analysis on vaccination trends in Section 3, showing the biggest drop in timely vaccine uptake at the 4 year event around the start of the pandemic.

Table 3 also shows that the results are robust to the inclusion of additional control variables to allow for observable differences in characteristics between the affected and the earlier-born cohort of children. In Panel B, we include covariates for region of residence, child's ethnicity, parental earnings level, parental marital status, and overseas-born parents. The results are very similar compared to the baseline effects, suggesting that the estimates of the effect of the pandemic are not driven by differences in characteristics between the groups.

Our estimates of pandemic-induced declines in childhood vaccination uptake for our affected cohorts of between zero and 15 pp are broadly in line with results from other retrospective cohort studies in high-income countries. These studies have found that on-time and/or up-to-date routine immunisation coverage among children aged under 5 years declined in the early months of the pandemic (up to May 2020, capturing lockdowns in many countries) relative to pre-pandemic levels by between 3 and 18 pp in US jurisdictions (Ackerson et al., 2021; Bode et al., 2021; Bramer et al., 2020; DeSilva et al., 2022), between 6 and 8 pp in the Netherlands (Middeldorp et al., 2021), between 0.5 and 1.9 pp in England (McQuaid et al., 2022), between 4 and 7 pp in Alberta, Canada (MacDonald et al., 2022), and between 1.7 and 14.7 pp in Ontario, Canada (Ji et al., 2022).

Our finding that uptake among the affected cohorts recovered to within 1 pp of pre-pandemic levels for most immunisation events (except the 4 year event which only partially recovered to 6 pp below pre-pandemic levels) is also broadly similar to experiences in other countries. From June 2020, vaccination coverage partially recovered in Ontario, Canada and Southern California, US to be 4 to 9 pp below pre-pandemic levels by the end of follow-up (Ackerson et al., 2021; Ji et al., 2022) and nearly completely recovered in the Netherlands and Alberta, Canada to 1 to 2 pp below pre-pandemic levels (MacDonald et al., 2022; Middeldorp et al., 2021).

Our finding that pandemic-induced declines in coverage were larger among older children (notably at the 4 year event) than among infants in our affected cohort is also borne out in the literature; studies have generally found that declines were smallest (or no decline was observed) among very young infants, were somewhat larger among children aged between 6 months and 2 years, and were largest among older children aged over 2 years (Ackerson et al., 2021; Bramer et al., 2020; Ji et al., 2022; Kujawski et al., 2022; MacDonald et al., 2022).

### 4.3 Child characteristics

In this and the following sections, we analyse potential heterogeneous effects of the pandemic on vaccine uptake, by estimating equation (1) separately for sub-samples of the affected cohort with different characteristics. We focus on the 4 year event, because Section 4.2 revealed the largest effects for this immunisation event.

**Fig. 3:**
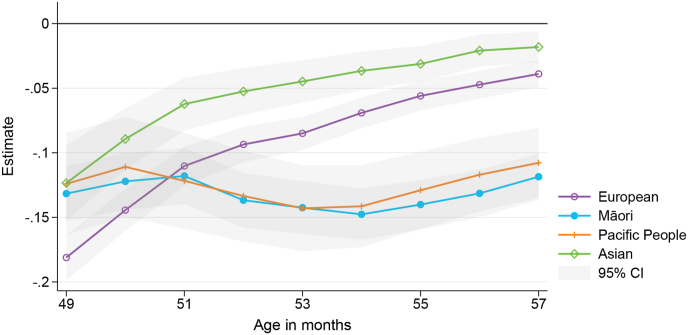
Effect of the COVID-19 pandemic on the 4 year immunisation event by ethnicity.

Fig. 3 shows the effect of the pandemic on the affected cohort for the 4 year immunisations when we split the sample by ethnicity, Table 4 shows the corresponding estimation output at age 49 and 57 months. The pandemic reduced timely vaccination among all ethnic groups. The share of fully immunised children at 49 months of age decreased between 12 and 18 pp. Interestingly, there are stark differences in catch-up behaviour during the following months. Vaccine uptake among European and Asian children almost converges to their earlier-born counterparts, with a 2 and 4 pp lower uptake by age 57 months. In contrast, the pandemic had a prolonged effect on Māori and Pacific children, whose vaccine uptake at age 57 months was still 12 and 11 pp lower compared to pre-pandemic levels.

The larger effects among Māori and Pacific children is alarming given that these children already had lower immunisation rates before the pandemic. Table 4 also reports mean uptake of unaffected children by ethnicity. It suggests that before the pandemic, the share of fully immunised children at age 57 months was 13 pp (Māori) and 6 pp (Pacific peoples) lower compared to Asian children, who had the highest immunisation rates. This is consistent with data reported by the Ministry of Health, which estimates that the share of fully immunised children at 5 years was 86.7 % and 91.1 % for Māori and Pacific children in the first quarter of 2020, compared to 93.8 % for Asian children (Ministry of Health, 2022). The COVID-19 pandemic has therefore led to a widening of existing inequalities.

In the US, there is conflicting evidence on whether ethnic inequalities widened as a result of the pandemic. Ackerson et al. (2021) find that the decline in coverage was larger, and recovery in coverage during the ‘reopening period’ of the pandemic weaker, among Black children in the US compared to non-Black children, but DeSilva et al. (2022) find that pre-pandemic Black vs. non-Black gaps in coverage were mostly preserved (not exacerbated) during the pandemic.

We also find large effect heterogeneity with respect to the birth order of children. Table 4 shows that there are large decreases in timely uptake of 14–17 pp for all children at the beginning of the pandemic at age 49 months. While first- and second-born children catch-up to a large extent over the following months, reducing the gap compared to earlier-born cohorts to 5 pp, uptake among third- and fourth-born children is still 9 and 14 pp lower. Past studies in other countries have also documented that birth order affects childhood vaccinations and have linked birth order differences in vaccination behaviour to parental resource constraints (Lin et al., 2022; Pruckner et al., 2021). In contrast, we find no marked differences between male and female children. Ji et al. (2022) also found no sex differences in the impact of the pandemic on coverage among Canadian children.

### 4.4 Parents’ socioeconomic status

**Fig. 4:**
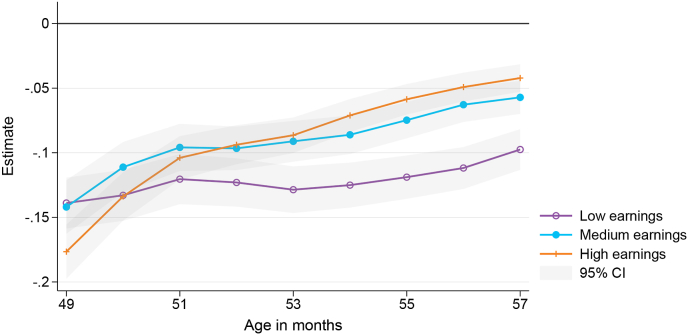
Effect of the COVID-19 pandemic on the 4 year immunisation event by parents' earnings.

Fig. 4 displays the effect of the pandemic on the 4 year immunisation event by parental earnings, Table 5 provides the corresponding estimation output at age 49 and 57 months. We find similar and large decreases in uptake at the onset of the pandemic for all earnings groups, but children in the affected cohort whose parents have high and medium earnings tend to catch-up faster and to a greater extent in the following months. At the end of the observation period (at age 57 months), vaccine uptake of children from families with low earnings is still 10 pp lower than pre-pandemic levels. There are similar differences when we stratify children by parents’ education level. Children with degree-qualified parents catch-up faster. By age 57 months, uptake among these children had recovered to 3 pp below pre-pandemic levels, but uptake among children whose parents do not have a degree was 9 pp below pre-pandemic levels.

An interesting finding is that the initial decrease is somewhat larger for families with high earnings and a high education level. Faster changes in vaccination behaviour connected to higher education have also been documented for the controversy linking the MMR vaccine to the development of autism (Anderberg et al., 2011) and measles outbreaks (Schober, 2020). A potential explanation is that parents with more education more quickly absorb and respond to new health-related information (Anderberg et al., 2011). In contrast, Ji et al. (2022) found the impact of the pandemic on coverage among Canadian children did not differ by neighbourhood income or neighbourhood socioeconomic deprivation.

We also find differences in the impact of the pandemic for further indicators of socioeconomic status (SES). Children in the affected cohort whose parents receive benefit payments did not seem to catch-up in terms of their vaccine uptake. At both the start and the end of the observation period, their vaccine uptake was 13 pp below pre-pandemic levels. We also find longer-lasting effects for children with unmarried parents, whose uptake at age 57 months is still 10 pp below pre-pandemic levels (compared to 4 pp lower for married). It is likely that parents in the unmarried group are more often sole parents. The differential impact may therefore be related to parental resources (including time) or other underlying differences. Regarding the birth place of the parents, we find somewhat larger effects for children whose parents were born in New Zealand compared to children who have at least one overseas-born parent. When we stratify by level of neighbourhood deprivation, we find similar patterns to when we measure socioeconomic status at the family level. While there are large effects at the beginning of the pandemic among all children, catch-up lags among those living in high deprivation areas; their uptake is still 10 pp below pre-pandemic levels, compared to effects of 4 pp for children living in low and medium deprivation areas.

### 4.5 Conditional effects

Given that many of the explanatory factors analysed, such as ethnicity and socioeconomic status, are correlated, the question arises as to how much of the variation in the impact of the pandemic between population subgroups is due to a particular characteristic, when other differences are controlled for. To investigate this, we estimate a variant of equation (1) that includes all examined characteristics as well as their interaction with the indicator of whether the child is in the affected cohort. Regression output for this full specification at the 4 year immunisation event is provided in Table A6 in Appendix A. For ease of comparison with the estimates of the unconditional effects for subgroups discussed above, Table 6 presents the corresponding average marginal effects of the pandemic for selected child and parental characteristics conditional on all other explanatory variables. They can be interpreted as effects for a particular group (e.g., ethnic group) allowing for differences in all other background characteristics included in the model (e.g., region of residence, parental earnings and education levels).

**Table 6:** Average marginal effects of the pandemic for selected child and parental characteristics on vaccine uptake at the 4 year immunisation event.

	49 months		57 months	
	Estimate	S.E.	Estimate	S.E.
	(1)	(2)	(3)	(4)
*Panel A: Ethnicity*				
European	−0.18***	(0.010)	−0.05***	(0.006)
Māori	−0.14***	(0.012)	−0.09***	(0.009)
Pacific People	−0.13***	(0.021)	−0.09***	(0.014)
Asian	−0.13***	(0.018)	−0.05***	(0.009)
*Panel B: Birth order*				
First born	−0.15***	(0.008)	−0.05***	(0.005)
Second born	−0.16***	(0.011)	−0.07***	(0.007)
Third born	−0.15***	(0.018)	−0.10***	(0.012)
Fourth born	−0.19***	(0.031)	−0.13***	(0.024)
*Panel C: Earnings*				
Low earnings	−0.15***	(0.011)	−0.07***	(0.008)
Medium earnings	−0.15***	(0.010)	−0.06***	(0.007)
High earnings	−0.16***	(0.012)	−0.07***	(0.007)
*Panel D: Benefit receipt*				
No benefit receipt	−0.16***	(0.007)	−0.06***	(0.005)
Benefit receipt	−0.15***	(0.014)	−0.09***	(0.011)
*Panel E: Education level*				
No degree	−0.15***	(0.008)	−0.07***	(0.005)
Bachelor's degree or higher	−0.16***	(0.010)	−0.06***	(0.006)
*Panel F: Marital status*				
Unmarried parents	−0.15***	(0.010)	−0.08***	(0.006)
Married parents	−0.15***	(0.009)	−0.06***	(0.006)

Notes: This table presents average marginal effects of the COVID-19 pandemic on the 4 year immunisation event derived from the full specification regression (Table A6) for different population subgroups. *p < 0.05, **p < 0.01, ***p < 0.001.

Comparing the unconditional findings in Table 5 to the conditional estimates in Table 6 we find that, once other factors are controlled for, the initial effects of the pandemic on 4 year immunisation uptake are similar within the subcategories of each of the various socioeconomic characteristics (parental earnings, benefit receipt, and education level). Previously, there appeared to be a larger initial impact for children in households with higher earnings, no benefit receipt and a higher educational level. However, after controlling for other child, parental and regional characteristics, the initial impact is similar regardless of whether low or high income household; or irrespective of benefit receipt status or educational level. Further, some of the differences in catch-up vaccination by parents’ earnings, education, and marital status are also no longer apparent in the conditional analysis.

In contrast, large differences between ethnic groups remain in the conditional analysis. The pandemic caused a 9 pp decrease in uptake among Māori and Pacific children by 57 months of age, compared to 5 pp for European and Asian children. These conditional effects are smaller than the unconditional effects discussed in Section 4.3 (−12 pp for Māori, −11 pp for Pacific, −4 pp for European, and −2 pp for Asian children), suggesting that differences in observed characteristics between ethnic groups account for some, but not all, of the heterogeneity in the pandemic's impact.

Differences in vaccination catch-up by birth order and parental beneficiary status also remain. Conditional on all other characteristics, vaccination catch-up is lower among children with higher birth order and of beneficiary parents. For example, the effect of the pandemic at nine months after eligibility is −5 pp for first-born children compared to −13 pp for fourth-born children.

## 5 Conclusion

This study examines the impact of the COVID-19 pandemic on paediatric vaccination coverage in New Zealand by comparing vaccine uptake of children who became eligible for immunisation during the pandemic to that of earlier-born cohorts. We find among our affected cohorts that the initial phase of the pandemic had, on average, small or nil effects on timely immunisation at the four infancy events (6 weeks, 3 months, 5 months, and 15 months) with declines in coverage of between zero and 3 pp compared to pre-pandemic levels, but a large effect at the 4 year event of −15 pp. Nine months after eligibility, catch-up among the affected cohorts was largely achieved for the infancy immunisations, but 4 year coverage remained 6 pp below pre-pandemic levels. The larger effect at 4 years may be related to flexible guidance issued by the Ministry of Health in late March 2020 that during the lockdowns the infancy immunisations should not be delayed, but that the 4-year immunisation event “potentially could be delayed for a short time, if necessary due to practice circumstances” (Ministry of Health and IMAC, 2020). Our findings indicate that this prioritisation was successful in maintaining infant immunisation levels at the start of the pandemic. But the importance of timely 4-year vaccinations should not be neglected. For example, existing evidence suggests waning immunity of infant pertussis vaccinations, and that preschool booster vaccination can not only decrease disease incidence in the targeted population but also reduce transmission to susceptible infants (Greeff et al., 2008; Zepp et al., 2011).

Timely uptake for the 4 year event initially dropped most among children of European ethnicity and of non-beneficiary parents but catch-up quickly surpassed their Māori, Pacific, and benefit-receiving counterparts for whom sizeable gaps in coverage below pre-pandemic levels remained at the end of our observation period. Across all other child and parental characteristics, initial impacts were fairly even, but catch-up lagged among children with third or fourth birth order and living in high deprivation neighbourhoods. Overall, the impact of the pandemic on immunisation coverage was small and negative among our affected cohorts of infants, large and negative among our affected cohort of 4-year-olds, and resulted in a widening of pre-existing ethnic and socioeconomic inequalities in immunisation coverage.

Healthcare avoidance or delay in seeking care (Imlach et al., 2022; Wilson et al., 2021) as well as postponement and cancellation of some services (Imlach et al., 2022) have been identified as reasons for pandemic-induced declines in childhood vaccine uptake in New Zealand. There is little empirical information on why the pandemic led to a widening of immunity gaps by ethnicity and socioeconomic status in New Zealand. However, it is known that Māori and Pacific peoples both in New Zealand and in the Pacific region have historically borne the heaviest burden of previous pandemics such as the 2009 H1N1 influenza pandemic and the 2019–2020 measles outbreak in New Zealand (Turner, 2019; Wilson et al., 2012). These past experiences may have made the Māori and Pacific populations more risk-averse with respect to COVID-19 exposure and hence more hesitant to get their children routinely vaccinated during the initial lockdown. Furthermore, Māori and Pacific peoples made up 84 % of cases in the August 2020 COVID-19 outbreak (Sonder et al., 2023) and their risk of hospitalisation for COVID-19 over the first seven months of the pandemic was 2.5–3.0 times greater than for other New Zealanders (Steyn et al., 2023), which may have had negative impacts on vaccination catch-up among Māori and Pacific children. Alternatively, lower access to the internet, digital devices, and other forms of digital exclusion may have reduced Māori and Pacific peoples’ exposure to public health communications from government and primary care practices including the message that routine immunisation services remained available during lockdowns (Ioane et al., 2021).

## Disclaimer

Access to the data used in this study was provided by Stats NZ under conditions designed to give effect to the security and confidentiality provisions of the Data and Statistics Act 2022. The results presented in this study are the work of the author, not Stats NZ or individual data suppliers. These results are not official statistics. They have been created for research purposes from the Integrated Data Infrastructure (IDI) which is carefully managed by Stats NZ. For more information about the IDI please visit https://www.stats.govt.nz/integrated-data/. The results are based in part on tax data supplied by Inland Revenue to Stats NZ under the Tax Administration Act 1994 for statistical purposes. Any discussion of data limitations or weaknesses is in the context of using the IDI for statistical purposes, and is not related to the data's ability to support Inland Revenue's core operational requirements. All observation counts are randomly rounded to base 3 in accordance with Stats NZ confidentiality rules.

## CRediT authorship contribution statement

**Leon Iusitini:** Writing – review & editing, Writing – original draft, Project administration, Methodology, Investigation, Funding acquisition, Formal analysis, Data curation, Conceptualization. **Gail Pacheco:** Writing – review & editing, Writing – original draft, Project administration, Methodology, Investigation, Funding acquisition, Formal analysis, Data curation, Conceptualization. **Thomas Schober:** Writing – review & editing, Writing – original draft, Project administration, Methodology, Investigation, Funding acquisition, Formal analysis, Data curation, Conceptualization.

## Declaration of competing interest

None.

## Data Availability

The data that has been used is confidential.

## Footnotes

1 The immunisation schedule relevant to our population of interest - children aged under five years - is provided in Table A1 in Appendix A.

2 Except at the 18 month milestone where DeSilva et al. (2022) find that the disparity in coverage between Black and other children increased during the pandemic.

3 We do not have information on the actual caregivers, and therefore implicitly assume that the biological parents are a good proxy for the socioeconomic status of a child's family. Additionally, we use the level of socioeconomic deprivation of a child's neighbourhood as a proxy for their socioeconomic status.

4 We keep the required vaccinations for each immunisation event constant, and label children as fully vaccinated if they have received the following vaccinations: 1 dose of 6-in-1, 1 dose of RV, 1 dose of PCV for the 6 week event. 2 doses of 6-in-1, 2 doses of RV, 1 dose of PCV for the 3 month event. 3 doses of 6-in-1, 2 doses of RV, 2 doses of PCV for the 5 month event. 3 doses of 6-in-1, 2 doses of RV, 3 doses of PCV, 1 dose of MMR, 1 dose of Hib, 1 dose of VV for the 15 month event. 3 doses of 6-in-1, 3 doses of PCV, 2 doses of MMR, 1 dose of Hib, 1 dose of DTaP-IPV for the 4 year event.

5 Note that at 58 months, children in the unaffected cohort born in May 2015 experience the start of the pandemic, which complicates the interpretation of any remaining differences.

6 The differences in the share of Asian children between cohorts are consistent with general population trends in New Zealand in recent years, where there has been a disproportionate increase in the Asian population (Stats NZ, 2020a).
