# Supplementary material for: Assessing the impact of the COVID-19 pandemic on childhood vaccine uptake with administrative data
Source: SSM Popul Health. 2024 Mar 28;26:101657. doi: 10.1016/j.ssmph.2024.101657 (PMC11002846; doi:10.1016/j.ssmph.2024.101657)
Supplement: Multimedia component 1 [file mmc1.pdf]

A Online Appendix

This Online Appendix provides additional material discussed in the manuscript "Assessing the impact of the COVID-19 pandemic on childhood vaccine uptake with administrative data".

Table A1: Vaccines for children under 5 years on New Zealand's National Immunisation Schedule (April 2018 to September 2020)

	DTaP-IPV-HepB/Hib	PCV10	RV1	MMR	Hib-PRP	VV	DTaP-IPV
6 weeks	•	•	•				
3 months	•	•	•				
5 months	•	•					
15 months		•		•	•	•	
4 years				•			•

Notes: DTaP-IPV-HepB/Hib = Diphtheria, tetanus, acellular pertussis, inactivated polio, hepatitis B and *Haemophilus influenzae* type b vaccine, PCV10 = 10-valent pneumococcal conjugate vaccine, RV1 = Rotavirus vaccine, Hib-PRP = *Haemophilus influenzae* type b polyribosylribitol phosphate vaccine, MMR = Measles, mumps and rubella vaccine, VV = Varicella vaccine, DTaP-IPV = diphtheria, tetanus, acellular pertussis and inactivated polio vaccine.

Table A2: Characteristics of affected and unaffected children at 15 month immunisation event (in %)

	(1) Covid-Affected	(2) Covid-Unaffected	(3) Difference	(4) p-value
Female	48.1	48.5	0.5	0.459
European	39.6	41.1	1.6	0.010
Māori	30.0	30.2	0.2	0.705
Pacific People	9.7	9.7	0.0	0.945
Asian	17.0	15.4	-1.6	0.001
Information on mother	98.9	99.2	0.3	0.012
Information on father	93.6	93.9	0.3	0.396
Characteristics of parents				
Born overseas	39.5	37.9	-1.5	0.010
Married	49.6	50.6	0.9	0.126
Benefit receipt	24.0	24.5	0.5	0.365
Bachelor's degree or higher	42.2	42.1	-0.1	0.866
Low earnings	33.1	34.6	1.4	0.013
Medium earnings	32.7	31.7	-1.0	0.095
High earnings	34.2	33.7	-0.5	0.410
Neighbourhood deprivation level				
Low deprivation	21.6	22.0	0.5	0.373
Medium deprivation	36.9	36.4	-0.4	0.493
High deprivation	41.6	41.5	-0.0	0.940

Notes: This table compares average characteristics of children affected (Column 1) and unaffected (2) by the pandemic. Column 3 shows the difference between groups, Column 4 shows the p-value testing the equality of the two means. The number of observations is 12,165 for parents' education level and 13,308 for all other characteristics.

Table A3: Characteristics of affected and unaffected children at 5 month immunisation event (in %)

	(1) Covid-Affected	(2) Covid-Unaffected	(3) Difference	(4) p-value
Female	49.0	48.5	-0.4	0.457
European	39.0	41.0	2.0	0.001
Māori	28.6	29.7	1.1	0.047
Pacific People	9.4	8.8	-0.6	0.087
Asian	19.1	16.9	-2.2	0.000
Information on mother	98.8	98.7	-0.1	0.380
Information on father	93.4	93.8	0.4	0.165
Characteristics of parents				
Born overseas	42.0	39.3	-2.7	0.000
Married	52.5	51.4	-1.1	0.079
Benefit receipt	20.7	22.5	1.8	0.000
Bachelor's degree or higher	44.1	43.1	-1.0	0.092
Low earnings	32.8	35.3	2.5	0.000
Medium earnings	33.4	32.7	-0.8	0.186
High earnings	33.8	32.0	-1.8	0.002
Neighbourhood deprivation level				
Low deprivation	21.3	20.9	-0.3	0.483
Medium deprivation	36.2	36.6	0.4	0.459
High deprivation	42.5	42.5	-0.1	0.888

Notes: This table compares average characteristics of children affected (Column 1) and unaffected (2) by the pandemic. Column 3 shows the difference between groups, Column 4 shows the p-value testing the equality of the two means. The number of observations is 12,369 for parents' education level and 13,536 for all other characteristics.

Table A4: Characteristics of affected and unaffected children at 3 month immunisation event (in %)

	(1) Covid-Affected	(2) Covid-Unaffected	(3) Difference	(4) p-value
Female	49.2	48.0	-1.2	0.059
European	38.5	39.6	1.1	0.064
Māori	29.2	29.9	0.7	0.215
Pacific People	9.9	9.7	-0.1	0.681
Asian	18.8	17.1	-1.7	0.000
Information on mother	98.8	98.8	0.1	0.641
Information on father	93.4	93.6	0.3	0.326
Characteristics of parents				
Born overseas	41.9	39.7	-2.2	0.000
Married	52.2	49.7	-2.4	0.000
Benefit receipt	21.9	23.9	2.0	0.000
Bachelor's degree or higher	42.7	42.3	-0.4	0.530
Low earnings	31.9	33.1	1.3	0.023
Medium earnings	33.0	32.6	-0.3	0.522
High earnings	35.2	34.2	-0.9	0.108
Neighbourhood deprivation level				
Low deprivation	20.4	20.8	0.3	0.468
Medium deprivation	36.1	36.9	0.8	0.140
High deprivation	43.5	42.3	-1.2	0.043

Notes: This table compares average characteristics of children affected (Column 1) and unaffected (2) by the pandemic. Column 3 shows the difference between groups, Column 4 shows the p-value testing the equality of the two means. The number of observations is 12,273 for parents' education level and 13,449 for all other characteristics.

Table A5: Characteristics of affected and unaffected children at 6 week immunisation event (in %)

	(1) Covid-Affected	(2) Covid-Unaffected	(3) Difference	(4) p-value
Female	48.8	48.1	-0.7	0.246
European	38.8	40.0	1.2	0.045
Māori	29.0	29.4	0.5	0.408
Pacific People	10.1	10.2	0.1	0.754
Asian	18.5	16.7	-1.8	0.000
Information on mother	98.8	99.0	0.2	0.105
Information on father	93.5	93.8	0.3	0.376
Characteristics of parents				
Born overseas	41.4	39.9	-1.5	0.013
Married	52.0	50.2	-1.8	0.002
Benefit receipt	22.3	23.7	1.3	0.009
Bachelor's degree or higher	42.8	42.7	-0.1	0.919
Low earnings	30.6	31.8	1.2	0.026
Medium earnings	32.9	32.6	-0.3	0.556
High earnings	36.5	35.6	-0.9	0.117
Neighbourhood deprivation level				
Low deprivation	13.7	13.6	-0.1	0.787
Medium deprivation	23.8	24.2	0.4	0.479
High deprivation	62.5	62.2	-0.3	0.666

Notes: This table compares average characteristics of children affected (Column 1) and unaffected (2) by the pandemic. Column 3 shows the difference between groups, Column 4 shows the p-value testing the equality of the two means. The number of observations is 12,570 for parents' education level and 13,797 for all other characteristics.

Table A6: Full specification regression results for the 4 year immunisation event

	49 months		57 months	
	Estimate (1)	S.E. (2)	Estimate (3)	S.E. (4)
A	−0.174***	(0.022)	−0.031*	(0.014)
Māori	−0.115***	(0.012)	−0.032***	(0.007)
Pacific People	−0.056**	(0.017)	0.021*	(0.010)
Asian	0.089***	(0.015)	0.043***	(0.007)
Other ethnicity	0.000	(0.028)	0.011	(0.015)
A × Māori	0.040*	(0.016)	−0.039***	(0.012)
A × Pacific People	0.047	(0.024)	−0.030	(0.016)
A × Asian	0.050*	(0.021)	0.009	(0.011)
A × Other ethnicity	0.086*	(0.040)	0.021	(0.023)
Female	−0.003	(0.008)	−0.007	(0.005)
A × Female	0.003	(0.012)	0.005	(0.008)
Second born	−0.041***	(0.009)	−0.001	(0.005)
Third born	−0.105***	(0.014)	−0.016	(0.008)
Fourth born	−0.118***	(0.024)	−0.016	(0.016)
Higher birth order	−0.232***	(0.027)	−0.095***	(0.023)
A × Second born	−0.009	(0.014)	−0.017	(0.009)
A × Third born	0.002	(0.020)	−0.042**	(0.014)
A × Fourth born	−0.039	(0.032)	−0.076**	(0.025)
A × Higher birth order	0.084*	(0.037)	−0.095**	(0.035)
Low earnings	−0.055***	(0.011)	−0.040***	(0.007)
High earnings	0.020	(0.011)	0.014*	(0.006)
A × Low earnings	0.000	(0.015)	−0.008	(0.011)
A × High earnings	−0.013	(0.016)	−0.008	(0.009)
Benefit receipt	−0.093***	(0.013)	−0.040***	(0.008)
A × Benefit receipt	0.009	(0.018)	−0.034*	(0.013)
Bachelor's degree or higher	0.024*	(0.010)	0.005	(0.006)
Missing education information	−0.051*	(0.021)	−0.070***	(0.017)
A × Bachelor degree or higher	−0.012	(0.014)	0.012	(0.009)
A × Missing education information	−0.004	(0.029)	0.009	(0.026)
Married	0.040***	(0.010)	0.009	(0.006)
A × Married	−0.002	(0.015)	0.026**	(0.009)
Born overseas	−0.005	(0.011)	0.010	(0.006)
A × Born overseas	0.017	(0.016)	−0.000	(0.010)
Northland Region	−0.093***	(0.023)	−0.085***	(0.018)
Waikato Region	−0.143***	(0.015)	−0.009	(0.009)
Bay of Plenty Region	−0.043*	(0.018)	−0.015	(0.012)
Taranaki Region	−0.047	(0.025)	−0.003	(0.016)
Manawatu-Wanganui Region	−0.038	(0.020)	−0.005	(0.013)
Wellington Region	0.007	(0.015)	0.015	(0.008)
Canterbury Region	−0.005	(0.014)	0.022**	(0.007)
Otago Region	0.054**	(0.021)	0.031**	(0.011)
Southland Region	0.084**	(0.028)	0.034*	(0.015)
Gisborne/Hawke's Bay	−0.124***	(0.021)	0.007	(0.013)
Tasman/Nelson/Malborough/West Coast	0.008	(0.024)	−0.003	(0.015)
Other/missing region	0.008	(0.307)	−0.257	(0.286)
A × Northland Region	0.009	(0.031)	0.003	(0.026)
A × Waikato Region	−0.032	(0.021)	−0.053***	(0.015)
A × Bay of Plenty Region	0.058*	(0.025)	0.017	(0.018)
A × Taranaki Region	0.056	(0.037)	0.019	(0.025)
A × Manawatu-Wanganui Region	0.064*	(0.028)	0.043*	(0.019)
A × Wellington Region	0.027	(0.021)	−0.005	(0.014)
A × Canterbury Region	0.003	(0.020)	0.027*	(0.011)
A × Otago Region	−0.019	(0.031)	0.002	(0.018)
A × Southland Region	−0.033	(0.042)	0.020	(0.024)
A × Gisborne/Hawke's Bay	−0.002	(0.028)	−0.020	(0.021)
A × Tasman/Nelson/Malborough/West Coast	0.094**	(0.034)	0.031	(0.022)
A × Other/missing region	−0.599	(0.308)	−0.594*	(0.287)
Low deprivation	0.014	(0.011)	0.016**	(0.006)
High deprivation	0.013	(0.010)	0.011	(0.006)
A × Low deprivation	−0.020	(0.016)	−0.017	(0.009)
A × High deprivation	−0.020	(0.014)	−0.031**	(0.010)
N	26,142		26,142	

Notes: Regression results for effects of the pandemic on the 4 year immunisation event at age 49 months (point estimate in column 2, robust standard error in column 3) and 57 months (columns 3 and 4). A is the indicator for being in the affected cohort. Regressions additionally controls for the child's calendar month of birth. * $p < 0.05$, ** $p < 0.01$, *** $p < 0.001$.
